# Effects of noise exposure and long working hours on oxidative stress levels and high-frequency hearing threshold damage in occupational populations

**DOI:** 10.3389/fpubh.2026.1827520

**Published:** 2026-05-07

**Authors:** Ping-Shan Jiang, Yan-Hua Li, Lu-Xi Bai, Xue-Yi Kuang, Yuan Zhao, Yong-Xiang Tang, Qing Li, Wen-Feng Zeng, Feng Zhu

**Affiliations:** 1 Occupational Health Surveillance Centre, The Affiliated Guangzhou Twelfth People's Hospital, Guangzhou Medical University, Guangzhou, China; 2 Occupational Health Management Section, The Affiliated Guangzhou Twelfth People's Hospital, Guangzhou Medical University, Guangzhou, China; 3 Central Laboratory, The Affiliated Guangzhou Twelfth People's Hospital, Guangzhou Medical University, Guangzhou, China

**Keywords:** combined exposure, high-frequency hearing loss, long working hours, noise, oxidative stress

## Abstract

**Introduction:**

Occupational noise-induced hearing loss remains a significant public health concern in China. This study aimed to investigate the associations between combined exposure to occupational noise and long working hours with oxidative stress levels and high-frequency hearing loss (HFHL) in an occupational population.

**Methods:**

A total of 547 Chinese workers from eight manufacturing enterprises in Guangzhou who were engaged in noise-exposed occupations were recruited. Participants were divided into a noise exposure group (weekly working hours ≤40 h, *n* = 332) and a combined exposure group (weekly working hours >40 h, *n* = 215). Pure-tone audiometry was performed, and serum levels of superoxide dismutase (SOD), catalase (CAT), malondialdehyde (MDA), and glutathione peroxidase (GPx) were measured using colorimetric microplate assays. Nonparametric tests and binary logistic regression analyses were conducted.

**Results:**

The study included 532 males (97.3%) and 15 females (2.7%). Compared with the noise exposure group, the combined exposure group presented significantly lower SOD activity and CAT activity (*p* < 0.05) and significantly higher MDA concentration (*p* < 0.05). The overall prevalence of HFHL was 43.14%, with significantly higher rates in the combined exposure group (*p* < 0.001). Logistic regression analysis revealed that the levels of SOD (OR = 0.869), CAT (OR = 0.963), and MDA (OR = 1.496) were significantly associated with HFHL. In forward stepwise multivariate analysis, only CAT remained an independent influencing factor (OR = 0.963, 95%CI: 0.941–0.985; *p* = 0.001).

**Conclusion:**

Combined exposure to noise and long working hours is associated with exacerbated oxidative stress and high-frequency hearing loss in occupational populations. CAT acts as an independent protective factor against HFHL, suggesting its potential as a biomarker for hearing damage assessment, although prospective validation is needed.

## Introduction

With the rapid development of industrialization, noise pollution has become a significant factor affecting human health, especially among occupational populations. Long-term exposure to high-noise environments poses a severe threat to employees’ auditory health. According to a survey of noise exposure among workers in the stamping workshop of an automobile manufacturing plant, the average noise exposure level during an 8-h workday was as high as 85 decibels, far exceeding the 8-h equivalent continuous noise exposure limit stipulated by China’s national occupational health standards (1). High-intensity noise exposure not only directly damages the auditory system but also exacerbates hearing loss through mechanisms such as oxidative stress and inflammatory responses (2). Additionally, long working hours are another important factor contributing to hearing loss. The 2023 report of the Ninth National Survey on the Status of Chinese Workers, conducted by the All-China Federation of Trade Unions, revealed that the average weekly working time for Chinese workers was 44.7 h, with 34.5% of workers working more than 45 h per week and 12.3% working more than 48 h per week (3). This long working pattern not only increases the duration of noise exposure but also intensifies oxidative stress in the body through pathways such as fatigue and decreased immunity, indirectly affecting auditory health (4).

Notably, the combined effect of noise and long working hours on hearing loss is not a simple additive effect. This interaction may be associated with the accumulation of oxidative stress, exacerbation of inflammatory responses, and neurodegenerative changes (5). In a study on the relationship between working hours and oxidative stress, the oxidative and antioxidant capacity of medical staff after working overtime on a 24-h shift schedule revealed that working overtime led to an imbalance in the antioxidant levels of medical staff. After working hours, the levels of oxidative stress markers in medical staff significantly increase (6). Working hours that exceed standard working hours stimulate the body to produce oxidative stress, triggering lipid peroxidation chain reactions, which in turn lead to oxidative DNA damage (7). Both noise and long working hours can affect the normal regulation of the hypothalamic–pituitary–thyroid (HPT) axis, thereby regulating mitochondrial function through the release of hormones, which affects the generation of reactive oxygen species (ROS) and oxidative stress (8, 9). These findings suggest that there may be interactions between noise and exposure duration, which together influence the biological effects of oxidative stress responses.

Occupational populations are potentially doubly threatened by high-noise environments and long working hours, which may severely impact employees’ auditory health. This study explored the cross-sectional associations between serum oxidative stress levels and high-frequency hearing loss in occupational populations exposed to both noise and long working hours by measuring relevant serum oxidative stress markers and conducting pure-tone audiometry among workers in Guangzhou, southern China.

## Methods

### Subjects

**Figure 1:**
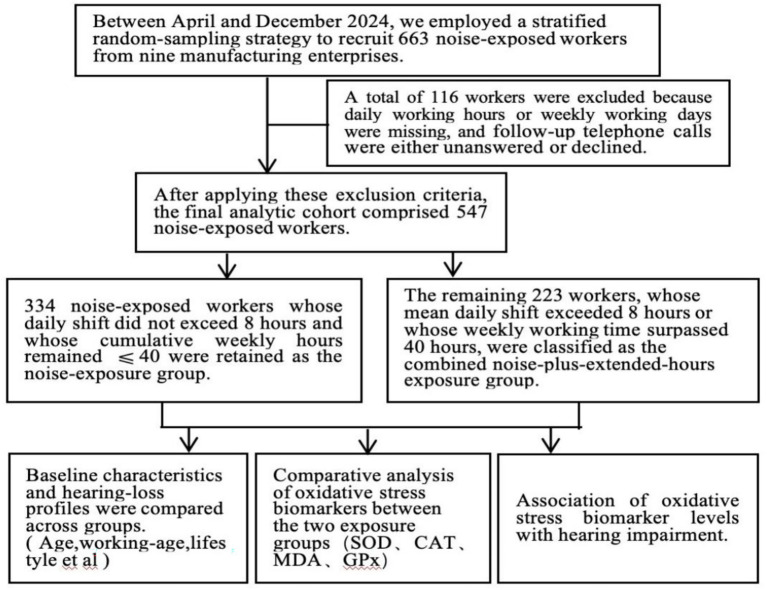
Flow diagram of participants selected for analyses in this study.

Between April and December 2024, a random sampling method was employed to select 547 workers engaged in noise-exposed occupations from eight manufacturing enterprises in Guangzhou, southern China, as the study subjects. The eight enterprises represented diverse industrial sectors: four automobile manufacturing companies (GAC Passenger Vehicle, GAC Toyota, Dongfeng Honda Engine, and ADVICS Auto Parts), one shipbuilding company (Yinghui Southern Shipbuilding), one glass manufacturing company (Asahi Glass Guangzhou), one aviation maintenance company (GAMECO), and one construction engineering company (Guangzhou Municipal Engineering General Contracting Branch). The inclusion criteria were as follows: (1) aged 18–60 years and (2) exposed to occupational noise hazards with a job tenure of ≥1 year and (3) provided informed consent. The exclusion criteria were as follows: (1) previous contraindications to noise-exposed occupations or a history of occupational diseases;(2) a family history of ear diseases or a history of ototoxic drug use; and (3) a previous history of otitis media, sensorineural hearing loss, or deafness. All participants provided informed consent, and all methods in this study were performed in accordance with the Declaration of Helsinki. The flow diagram of the participants selected for the analyses in this study is shown in Figure 1.

### Questionnaire survey

A self-designed questionnaire was used. Members of the research team, who had undergone unified training, guided the subjects in completing the online survey questionnaires onsite. General information from the subjects, including age, duration of exposure to occupational hazards, smoking status, alcohol consumption, physical exercise, daily working hours, weekly working days, and protective measures, was collected. On the basis of their working hours, the subjects were divided into two groups: the noise exposure group, comprising 334 subjects exposed to noise with a weekly working time not exceeding 40 h, and the combined exposure group (hereinafter referred to as the combined exposure group), comprising 223 subjects exposed to noise with a weekly working time exceeding 40 h.

### Determination of oxidative stress markers

Five milliliters of fasting venous blood was collected from the study subjects. After the samples were allowed to acclimate at room temperature for 30 min, they were centrifuged at 3,500 rpm for 10 min to obtain serum. The samples were stored at −20 °C and transported to Jiangsu Adison Biotechnology Co., Ltd., for unified detection. All markers were measured using colorimetric microplate assays:

MDA (malondialdehyde):thiobarbituric acid (TBA) method: The detection range was 2.92–40 nmol/mL; the sensitivity was 1.13 nmol/mL; the intra-assay CV was 4.1%; and the interassay CV was 7.2%.
SOD (superoxide dismutase): WST-8 method, wavelength 450 nm. CAT (catalase): Visible spectrophotometry, wavelength 510 nm.
GSH-Px (glutathione peroxidase): Colorimetric method, wavelength 412 nm.
Quality control: All the assays were performed in duplicate, with standard curves generated for each batch.

### Pure tone audiometry method

Pure-tone audiometry was conducted by professional occupational health physicians who had received training in occupational health regulations and had extensive experience. Testing was performed in accordance with the “Diagnostic Criteria for Occupational Noise-Induced Hearing Loss” (GBZ 49—2014) (10). Workers underwent pure-tone audiometry in a soundproof room with a background noise level of less than 25 dB(A) after being removed from the noisy environment for 24 h. Background calibration was performed during the testing process. Using a Madsen Itera audiometer (Madsen, Denmark), pure-tone air conduction audiometry was conducted for both ears at frequencies of 500 Hz, 1,000 Hz, 2000 Hz, 3,000 Hz, 4,000 Hz, and 6,000 Hz for all the study subjects. In this study, which focused on high-frequency hearing threshold abnormalities, the results of pure-tone air conduction audiometry at 3000 Hz, 4,000 Hz, and 6,000 Hz for both ears were selected for analysis.

### Criteria for hearing abnormality determination

The air-conduction hearing thresholds of the subjects were adjusted for age and sex in accordance with the “Statistical Distribution of the Relationship between Hearing Threshold and Age” (GBZ 7582—2004) (11). High-frequency hearing loss was defined as a pure-tone air-conduction hearing threshold greater than 25 dB at any one of the frequencies 3,000 Hz, 4,000 Hz, or 6,000 Hz in either ear. This definition captures the characteristic “noise notch” pattern at 4000–6000 Hz typical of early noise-induced hearing loss.

### Criteria for determining long working hours

In accordance with the “Labor Law of the People’s Republic of China” (12), the national working hours system stipulates that employees work 8 h per day and an average of 40 h per week. Workers with an average daily working time exceeding 8 h or a total weekly working time exceeding 40 h were classified as having long working hours.

### Quality control

This study was approved by the Ethics Committee of our hospital (Approval No. 2023072). Written consent was obtained from all participants, all participants provided informed consent, and all methods in this study were performed in accordance with the Declaration of Helsinki. Trained research staff guided the workers through the completion of the questionnaires, which were recorded in real time via an online survey platform. The occupational health physicians involved had received training in relevant occupational health regulations and held the necessary certification. All the instruments and equipment used were regularly calibrated and were within the calibration validity period. The participants were tested after a 24-h period of noise-free exposure, and the test results were subsequently adjusted for age and sex.

### Statistical analysis

Quantitative data are expressed as medians with interquartile ranges M (P25–P75) and were compared using the Wilcoxon rank-sum test. Categorical data are presented as *n* (%) and were compared using the chi-square (*χ*^2^) test or Fisher’s exact test. Univariate logistic regression was conducted for oxidative stress markers. Variables with *p* < 0.05 were included in multivariate logistic regression with forward (LR) stepwise selection (entry criterion *p* ≤ 0.05, removal criterion *p* ≥ 0.10). The multivariate model was adjusted for age, sex, smoking status, alcohol consumption, exercise habits, years of exposure, and noise intensity. Odds ratios (ORs) and 95% confidence intervals (CIs) were calculated. Model fit was assessed using the Hosmer–Lemeshow test. Significance level: *α* = 0.05 (two-tailed).

## Results

### Basic characteristics

Among the 547 study subjects, all were exposed to noise hazards according to the provided test reports. A total of 532 were male (97.3%), and 15 were female (2.7%). There was no significant difference in sex distribution between groups (*p* = 0.772 by Fisher’s exact test). The median age was 29.0 years (P25–P75: 26.0, 36.0), the median exposure duration was 6.92 years (3.33, 11.00), and the median noise exposure intensity was 84.1 dB (A) (80.7, 86.1). No statistically significant differences in age, exposure duration, noise intensity, smoking status, alcohol consumption, or exercise habits were detected between the groups (*p* > 0.05) (Table 1).

**Table 1:** General conditions of the occupational population in the noise and combined exposure groups.

Variable	Noise exposure group (*n* = 332)	Combined exposure group (*n* = 215)	*Z/χ* ^2^	*p*
Age (years)	29 (26.0,36.0)	30 (27.0,36.0)	−1.544	0.123
Years of exposure (years)	6.46 (3.3,11)	7 (3.5,10.9)	−1.227	0.220
Noise intensity [dB(A)]	84.1 (80.7,86.2)	83.3 (80.6,85.8)	−0.664	0.507
Sex, *n* (%)			—	0.772^*^
Male	321 (96.7)	211 (98.1)		
Female	11 (3.3)	4 (1.9)		
Smoking, *n* (%)			3.322	0.345
Never smoked	164 (49.4)	91 (42.3)		
Occasional cigarette	38 (11.4)	21 (9.8)		
Regular smoking	118 (35.5)	90 (41.9)		
Have given up smoking	12 (3.6)	13 (6.0)		
Alcohol consumption, *n* (%)			5.151	0.161
Never drink alcohol	161 (48.5)	90 (41.9)		
Occasional drinking	146 (44.0)	102 (47.4)		
Regular consumption of alcohol	9 (2.7)	3 (1.4)		
Sober	16 (4.8)	20 (9.3)		
Exercise habit, *n* (%)			0.238	0.888
Never worked out	43 (13.0)	28 (13.0)		
An occasional workout	244 (72.9)	157 (73.0)		
Regular exercise	47 (14.1)	30 (14.0)		

* Fisher’s exact test.

### Comparison of oxidative stress levels between the noise-exposed group and the combined exposure group in occupational populations

Serum SOD activity and CAT activity were significantly lower in the combined exposure group than in the noise exposure group (*p* < 0.05), and the serum MDA concentration was significantly greater in the combined exposure group than in the noise exposure group (*p* < 0.05) (Table 2).

**Table 2:** Comparison of oxidative stress markers between the noise exposure and combined exposure groups.

Variable	Noise group M (P25-P75)	Combined group M (P25-P75)	*Z/χ* ^2^	*p*
SOD (U/mL)	7.09 (6.45, 8.04)	7.03 (6.33, 7.80)	−1.76	0.039
CAT (μmol/min/mL)	21.89 (17.58, 26.77)	20.31 (16.22, 25.22)	−3.11	0.002
MDA (nmol/mL)	3.48 (2.85, 4.20)	3.55 (3.04, 4.25)	−2.06	0.039
GPx (nmol/min/mL)	182.51 (158.52, 208.68)	184.74 (157.99, 212.39)	−0.05	0.958

### High-frequency hearing threshold damage in occupational populations exposed to noise and combined exposure

Among the 547 study subjects, 236 individuals had high-frequency hearing threshold damage, for a detection rate of 43.14%. The rate of high-frequency hearing threshold damage was significantly greater in the combined exposure group than in the noise-exposed group (*p* < 0.05) (Table 3).

**Table 3:** Prevalence of high-frequency hearing loss in occupational populations.

Group	*n*	HFHL cases	Prevalence (%)	*χ* ^2^	*p*
Overall	547	236	43.14	—	—
Noise exposure group	332	105	31.63	38.47	<0.001
Combined exposure group	215	131	60.93		

Significant differences in hearing threshold damage rates at 4000 Hz and 6,000 Hz were detected between the two groups in both the left and right ears (*p* < 0.05). A characteristic noise-notch pattern (4,000 Hz and 6,000 Hz damage rates exceeding 3,000 Hz) was observed in both groups, with more pronounced high-frequency damage in the combined exposure group (Table 4).

**Table 4:** High-frequency hearing loss rates by ear and frequency.

Frequency	Ear	Noise group [n/N (%)]	Combined group [n/N (%)]	*χ* ^2^	*p*
3,000 Hz	Left	29/332 (8.73)	39/215 (18.14)	9.94	0.002
	Right	21/332 (6.33)	33/215 (15.35)	10.36	0.001
4,000 Hz	Left	48/332 (14.46)	54/215 (25.12)	8.42	0.004
	Right	39/332 (11.75)	48/215 (22.33)	11.22	0.001
6,000 Hz	Left	59/332 (17.77)	73/215 (33.95)	18.48	<0.001
	Right	55/332 (16.57)	79/215 (36.74)	26.01	<0.001

### Analysis of factors influencing high-frequency hearing threshold damage

Univariate logistic regression analysis revealed that SOD (OR = 0.869; 95%CI: 0.760–0.995; *p* = 0.042), CAT (OR = 0.963; 95%CI: 0.941–0.985; *p* = 0.001), and MDA (OR = 1.496; 95%CI: 1.156–1.937; *p* = 0.002) were significantly associated with HFHL (Table 5).

**Table 5:** Univariate logistic regression analysis of oxidative stress markers associated with high-frequency hearing loss.

Variable	B	SE	Wald *χ*^2^	*p*	OR	95% CI
SOD (U/mL)	−0.140	0.068	4.26	0.042	0.869	0.760–0.995
CAT (μmol/min/mL)	−0.038	0.012	10.81	0.001	0.963	0.941–0.985
MDA (nmol/mL)	0.403	0.131	9.44	0.002	1.496	1.156–1.937
GPx (nmol/min/mL)	−0.001	0.003	0.10	0.749	0.999	0.993–1.005

In forward stepwise (LR) multivariate analysis adjusted for confounders, only CAT remained in the final model (OR = 0.963, 95%CI: 0.941–0.985; Wald *χ*^2^ = 10.81; *p* = 0.001). The model fit was good (Hosmer–Lemeshow *χ*^2^ = 14.78, df = 8, *p* = 0.064) (Table 6).

**Table 6:** Multivariate stepwise logistic regression analysis of factors associated with high-frequency hearing loss.

Variable	B	SE	Wald *χ*^2^	*p*	OR	95% CI
CAT (μmol/min/mL)	−0.038	0.012	10.81	0.001**	0.963	0.941–0.985
Constant	0.576	0.269	4.58	0.032	1.779	—

The forward stepwise (likelihood ratio) method was used. Candidate variables included SOD, CAT, MDA, and GPx. Only CAT met the entry criterion (*p* < 0.05) and remained in the final model. Model fit: Hosmer–Lemeshow test, *χ*^2^ = 14.78, df = 8, *p* = 0.064 (good fit).

## Discussion

According to relevant statistics, approximately 80 million workers in China are currently exposed to noise levels exceeding occupational limits (13). In recent years, the incidence of occupational noise-induced hearing loss in China has been increasing, and it has become the second most common occupational disease after pneumoconiosis (14). Our study revealed that the detection rate of high-frequency hearing threshold abnormalities via pure-tone audiometry among occupational populations in Guangzhou, southern China, was 43.14%, which was slightly higher than the rates of 40.53 and 35.26% reported previously (11, 15). This rate is also significantly higher than the 18.89% hearing abnormality detection rate reported previously (16) in the automotive manufacturing industry, where the high-frequency hearing abnormality detection rate was 18.04%. The differences in high-frequency hearing threshold abnormality detection rates are attributed primarily to the comprehensive occupational protection measures in place in the automotive manufacturing industry in Guangzhou, as well as the timely reassignment of workers upon detection of hearing loss, which may have led to an underestimation of the extent of hearing damage caused by noise.

Through pure-tone audiometry, this study compared high-frequency hearing threshold damage in individuals exposed to noise and those exposed to a combination of noise and long working hours in Guangzhou, southern China. The results revealed an increased rate of high-frequency hearing threshold damage in the combined exposure group, primarily manifested as hearing threshold damage at 4000 Hz and 6,000 Hz in both ears. These findings are consistent with the results reported that early noise-induced hearing loss is initially characterized by elevated hearing thresholds at 4000 Hz and 6,000 Hz (17)^,^ which is consistent with the early “noise notch” pattern characteristic of noise-induced hearing loss (18). These findings suggest that combined exposure to noise and long working hours may exacerbate early noise-induced hearing damage patterns.

Long-term occupational noise exposure induces cochlear oxidative stress—characterized by suppressed activity of antioxidant enzymes (SOD, GPx, and CAT) (19) and elevated malondialdehyde(MDA) levels (20), leading to reactive oxygen species accumulation and subsequent hair cell damage that manifests as hearing impairment. Additionally, extended working hours may exacerbate oxidative damage through hypothalamic–pituitary–adrenal (HPA) axis activation and sustained cortisol elevation, which further attenuate antioxidant defenses and promote mitochondrial reactive oxygen species release (21, 22). In this study, the effects of combined exposure to noise and long working hours on oxidative stress levels were compared with those of noise exposure alone. The results revealed that in Guangzhou, southern China, compared with the noise exposure group, the combined exposure group presented lower SOD and CAT activities and higher MDA concentrations. These findings are consistent with the effects of noise or long working hours on oxidative stress reported previously (19–22). Combined exposure to noise and long working hours induces a more pronounced oxidative stress response, characterized by the suppression of endogenous antioxidant enzyme activity (SOD and CAT activity), intensified systemic oxidative stress, and excessive free radical production (such as ROS). These free radicals attack polyunsaturated fatty acids (PUFAs) in cell membranes, triggering lipid peroxidation chain reactions and subsequently producing excessive amounts of MDA. These findings suggest that combined exposure to noise and long working hours may involve synergistic mechanisms that exacerbate oxidative stress, which have yet to be discovered. Compared with single-factor exposure, combined exposure to noise and long working hours increases the degree of oxidative stress caused by single-factor exposure, resulting in more severe oxidative stress and hearing damage to the human body. The mechanism may involve both noise and long working hours, leading to a decrease in the mitochondrial membrane potential (23, 24) and reduced ATP production. Combined exposure may accelerate mitochondrial dysfunction, causing more severe oxidative damage. Additionally, noise activates protein kinase C via the renin–angiotensin system (25), upregulates NOX_2_ expression, and leads to excessive ROS in blood vessels and brain tissue. Long working hours induce chronic stress (23), further promoting NOX_2_-dependent oxidative stress. Moreover, inflammatory factors induced by noise (such as IL-6 and CRP) form a positive feedback loop, with elevated cortisol levels associated with long working hours (24, 25), exacerbating oxidative stress and causing more severe hearing impairment. However, given the cross-sectional design, we cannot determine whether oxidative stress preceded hearing loss or resulted from it.

In subsequent studies, we conducted univariate logistic regression analyses using SOD, CAT, and MDA levels as independent variables and high-frequency hearing threshold damage as the dependent variable. The results indicated that SOD, CAT, and MDA are factors that influence high-frequency hearing threshold damage, with SOD and CAT acting as protective factors and MDA acting as a risk factor. A review on the prevention and treatment of noise-induced hearing loss through antioxidant stress revealed that the activity of SOD and CAT, which are antioxidant enzymes, may exacerbate oxidative stress damage when their levels decrease, whereas the level of MDA, a product of lipid peroxidation, reflects the degree of oxidative damage. All three genes are associated with hearing loss, which is consistent with our findings (26).

According to the logistic regression analysis, SOD and CAT were protective factors against HFHL, whereas MDA was a risk factor. After adjusting for potential confounders, only CAT remained an independent influencing factor (OR = 0.963, *p* = 0.001). For every one-unit increase in CAT activity, the odds of HFHL decreased by approximately 3.7%. These findings are consistent with the role of CAT as a key antioxidant enzyme that catalyzes the decomposition of hydrogen peroxide to water and oxygen, thereby protecting cochlear hair cells from oxidative damage (27). However, these cross-sectional findings require prospective validation before CAT can be established as a clinical biomarker.

## Limitations

This study has several limitations. First, the cross-sectional design precludes the establishment of causal relationships or temporal sequences between exposures and outcomes. Second, noise exposure was assessed through workplace fixed-point monitoring rather than individual dosimetry, potentially introducing exposure misclassification. Third, working hours were self-reported, which may be subject to recall bias. Fourth, the lack of a non-noise-exposed control group limits our ability to isolate the specific effects of noise from other occupational hazards. Fifth, the small number of female participants (*n* = 15) limits generalizability to female workers. Sixth, oxidative stress markers were measured at a single time point, which may not reflect cumulative exposure effects. Finally, although we adjusted for major confounders, residual confounding from unmeasured variables cannot be excluded.

## Conclusion

Combined exposure to noise and long working hours is associated with exacerbated oxidative stress and an increased prevalence of high-frequency hearing loss in occupational populations. Catalase acts as an independent protective factor against HFHL, suggesting its potential as a biomarker for hearing damage assessment, although prospective validation is needed. These findings suggest that reducing working hours and enhancing antioxidant defenses might be beneficial for preventing occupational hearing loss, but causal inferences require confirmation through longitudinal studies.

## Data Availability

The original contributions presented in the study are included in the article/supplementary material, further inquiries can be directed to the corresponding authors.
